# Antibacterial Activity of Diode Laser and Sodium Hypochlorite in *Enterococcus Faecalis*-Contaminated Root Canals

**DOI:** 10.7508/iej.2016.01.002

**Published:** 2015-12-24

**Authors:** Khosrow Sohrabi, Aidin Sooratgar, Kaveh Zolfagharnasab, Mohammad Javad Kharazifard, Farzaneh Afkhami

**Affiliations:** a *Department of Endodontics, Dental School, Tehran University of Medical Sciences, International Campus, Tehran, Iran****; ***; b *General Dentist, Tehran, Iran****; ***; c *Department of Epidemiology, Tehran University of Medical Sciences, International Campus, Tehran, Iran*

**Keywords:** Diode Laser, *Enterococcus faecalis*, Root Canal Disinfection, Root Canal Irrigant

## Abstract

**Introduction:**

The aim of the present *in vitro* study was to evaluate the disinfection ability of 980-nm diode laser in comparison with sodium hypochlorite (NaOCl) as a common root canal irrigant in canals infected with *Enterococcus faecalis* (*E. faecalis*).

**Methods and Materials:**

The root canals of 18 extracted single-rooted premolars were prepared by rotary system. After decoronation, the roots were autoclaved. One specimen was chosen for the negative control, and the remaining teeth were incubated with *E. faecalis* suspension for two weeks. Subsequently, one specimen was selected as the positive control and the remaining samples were divided into two groups (*n*=8). The samples of the first group were irrigated with 5.25% NaOCl and the second group were treated with a 980-nm diode laser. Microbial samples were taken from the root canals and bacterial cultivation was carried out. The average value and the standard deviation of colony-forming units (CFU) of each specimen were measured using descriptive statistics. The student’s t-test was used to compare the reduction in CFU in each group. The equality of variance of CFU was measured by the Levene’s test.

**Results:**

NaOCl resulted in 99.87% removal of the bacteria and showed significantly more antibacterial effect compared to the 980-nm diode laser which led to 96.56% bacterial reduction (*P*<0.05).

**Conclusion:**

Although 5.25% NaOCl seems to reduce *E. faecalis *more effectively, the diode laser also reduced the bacterial count. Therefore a 980-nm diode laser could be considered as a complementary disinfection method in root canal treatment.

## Introduction

The main purpose of root canal treatment is to eliminate the microorganisms and their by-products from the root canal and its tubular system, and also to prevent the re-entry of the microorganisms into the root canal system [1]. Using mechanical methods is incapable of thoroughly cleaning this complex tubular system by itself [2, 3]. When the bacterial infection occurs in the root canal system and the bacteria penetrate into the deep dentinal layers, cleaning of the dentinal tubules of the root canal becomes more difficult [4].

Many irrigants have been utilized with the mechanical methods in order to thoroughly clean the root canal system. The existing irrigants remove microorganisms through direct contact with them and are thus unable to penetrate deep into the dentinal tubules and clean them [5, 6]. Sodium hypochlorite (NaOCl) is the most commonly used endodontic irrigant. Many investigations have demonstrated the efficacy and the advantages of NaOCl [7-9] but the problems arising from its high tissue toxicity [10-13] and low penetration depth into the dentinal tubules [5, 14] have resulted in reduced desire to apply it.

*Enterococcus faecalis* (*E. faecalis*) is the most common bacterial species in resistant or recurrent infections leading to endodontic treatment failures [15, 16]. This cocci can resist the antibacterial agents such as NaOCl through different mechanisms [17, 18]. *In vitro* studies showed that *E. faecalis* can form biofilm and invade dentinal tubules even under stressed conditions [19, 20]. It is also resistance to alkaline pH and consequently to calcium hydroxide pastes, that normally inhibits other bacteria. The related mechanism may be related to the existence of a working active proton pump in the cellular membrane of this bacteria [21].

Considering the weaknesses of common irrigants in root canal treatment, in recent years new methods such as lasers have been introduced in order to effectively clean the root canal system. Among different types of the lasers, the diode laser is the most desirable type due to the properties such as high penetration depth into the dentinal tubules and proper antibacterial effect [22, 23]. Therefore, the present *in vitro* study was performed to compare the disinfection ability of 980-nm diode laser, to that NaOCl in endodontic treatment of teeth infected with *E. faecalis*.

## Materials and Methods

***Sample preparation***

This study was performed on 18 single-rooted, single-canal, intact mandibular premolars. Crowns of all teeth were cut off at cementoenamel junction using a high-speed handpiece and the root lengths were standardized to a 15 mm length.

All the root canals were prepared using the ProTaper rotary system (Dentsply Maillefer, Ballaigues, Switzerland) with crown-down technique up to F3 (30/0.09). Then 5.25% NaOCl and 17% ethylenediaminetetraacetic acid (EDTA) (Dia-Prep Plus, Diadent Group International Inc., Chongju, Korea) were used to remove the smear layer. The apical foramen was sealed using a self-cure glass-ionomer (Densell, Dental Medrano SA, Buenos Aires, Argentina) and the external surfaces of the teeth were covered with two layers of colorless varnish to prevent liquid penetration.

In order to sterile the samples, each tooth was placed in a cryo-tube (Cryo. S, PP Greiner Bio-One Gmbh., Frickenhausen, Germany) containing sterile brain-heart infusion (BHI) agar (Difco Laboratories, Detroit, MI, USA) and were autoclaved under operating conditions of 121^°^C and 15 psi pressure for 30 min.

One tooth was randomly selected as the negative control which was placed in an incubator for 2 weeks. Then 50 µL of *E. faecalis* suspension (ATCC 29212) was inoculated in 5 mL of BHI and was kept in an incubator at 37^°^C for 24 h. The concentration of the test suspension was then adjusted to 0.5 McFarland standard turbidity (1.5×10^8^ CFU/mL). Under sterile condition, each tooth was brought out of the tube containing BHI and its root canal was dried using sterile paper points (Gapadent Co Ltd, Tianjin, China).

About 0.01 mL standard value of the bacterial suspension was then placed in to each root canal. Thereafter each sample was placed in a new tube containing BHI and was stored at 37^°^C for two weeks. During this period, the intra-canal suspension was replaced with 0.01 mL of a new suspension every 48 h.

***Root canal disinfection***

After two weeks, one tooth among the samples was randomly served as the positive control and the residual teeth were randomly submitted to two groups of eight (*n*=8): In the first group, the BHI broth inside the root canals was dried using sterile paper points before irrigation with 5 mL of 5.25% NaOCl (Golrang, Pakshoo Co. Tehran, Iran) for 5 min. Then, the canals were dried using paper points and 10 µL of BHI broth was again placed in the canals. Each tooth was finally placed in a sterile cryo-tube and was stored in an incubator at 37^°^C for 24 h.

In the second group, intra-canal BHI broth was dried using sterile paper points. Laser irradiation procedure was performed five times for five sec each time, with a 15 sec interval. 

For the laser treatment protocol a diode laser [gallium-aluminum-arsenate laser (GaAIAs)] (Deka Laser Technologies Inc. CA, USA) was applied with the wave length of 980-nm, output power of 2.5 W, continuous wave mode, 10-m sec pulse duration and 10-m sec pulse interval. The laser was irradiated into the canals to a depth of 1 mm shorter than the actual root canal length, using an optical fiber with a diameter of 320 µm. The handpiece had an angle of approximately 10 degrees with the root canal wall. During the time of lasing, the handpiece was moved in a circular motion from the apical towards the coronal part (step-back technique), without cooling system.

When laser treatment was done, 10 µL of BHI broth was placed into the canals. Then each tooth was put in a sterile cryo-tube and was kept in an incubator at 37^°^C for 24 h, similar to the previous group.

The root canals in the positive control group was dried using sterile paper points and was then irrigated with 5 mL of normal saline solution. 

***Root canal sampling***

After 24 h the BHI broth inside all the canals was dried using sterile paper points and the canals were refilled with sterile normal saline as a transfer fluid. Sampling from the dentin of all root canals in each group was performed by filing the canals for 20 sec, using #15 and #25 Hedstrom files (Mani, Tochigi, Japan) in the apical and coronal zones, respectively. The transfer fluid and dentin chips inside each canal were then collected applying sterile paper points.

The files and the paper point of each tooth were placed in a test tube containing 10 mL of normal saline. The test tube was then shaken in vortex mixer for 20 sec. Finally, 50 µL of vortexed normal saline was inoculated in a plate containing blood agar. 

Following incubation at 37^°^C for 48 h, the number of colony-forming units (CFU) was determined by a colony counter (Colony Star, Funke Gerber Product, Gebr Liebisch, Germany).

The preliminary analysis with the Kolmogrof-Smirnov test confirmed normal distribution of the data. The average value and the standard deviation of CFU of each specimen were measured using descriptive statistics. The student’s t-test served to evaluate the significant reduction in the CFU in each group in comparison with the positive control group. The equality of variance of CFU was measured by the Levene’s test. The level of significance was set at 0.05.

## Results

No bacterial growth occurred in the negative control group. The average CFU in the positive control sample was 1.18×10^4^±8.1×10^3^. Laser irradiation reduced the average number of CFU to 405.37±395.08 CFU/mL while NaOCl reduced the average CFU to 14.5±3.66 CFU/mL (Table 1).

The Levene's test revealed a statistically significant difference (*P*=0.002) in the variance of CFU between the 980-nm laser group and the NaOCl group. Additionally, the t-test showed a statistically significant difference between the two groups (*P*=0.027). According to the results of these tests, following the disinfection protocol, a significant difference between the number of colonies in the NaOCl group (mean±SD=14.50±3.66) and the laser group (mean±SD=405.37±395.08) was observed (*P*=0.027).

## Discussion

The present study compared the efficacy of a 980-nm diode laser with NaOCl, in removing *E. faecalis* from the root canal system. Presence of bacteria within the complex network of the root canal system and the dentinal tubules is considered to be the most important reason for endodontic treatment failure [1, 4]. Thus, complete elimination of bacteria and their toxins is the fundamental of a successful endodontic treatment.

**Table 1:** The mean (SD) of colony-forming units per mL (CFU/mL) in two experimental and positive control groups

**Group**	**Mean (SD) **	**Disinfection (%)**
**Positive control **	1.18×10⁴ (8.1×10^3^)	21.33
**NaOCl **	14.5 (3.66)	99.87
**Laser **	405.37 (395.08)	96.56

During root canal preparation using chemomechanical approach, the superficial layers of dentin and a part of infected pulp tissue can be removed; moreover, the effect of chemical irrigants is limited to the most superficial layers of the root dentin [2, 3, 5, 6].

Many investigations have used *E. faecalis* to evaluate the disinfection potential of antibacterial agents or various kinds of lasers [24-27]; this cocci is highly resistant to many disinfecting agents and also is particularly important in persistent endodontic infections and failed root canal treatments [17, 28, 29]. On the other hand, since the main antibacterial effect of the laser is principally a result of the heating effect [30] and* E. faecalis* is very heat-resistant [17, 28, 29], it is recommended to be used to evaluate the heat-independent antimicrobial effect of laser. 

In order to compare the results with the previous investigations, a similar design with minimal differences in microbiologic methods and the diode laser settings was used. In this study, the laser irradiation protocol was selected based on the factory setting and the samples were irradiated for 25 sec (5 times of irradiation with 5 sec durations and 15 sec intervals) with the output power of 2.5 W, frequency of 50 Hz and continuous wave mode. Similar to other studies by Ashofteh *et al.* [24] and Kanumuru *et al.* [25], in the present study the optical fiber of the laser was directly inserted into the root canal. Thus, the fiber tip was in direct contact with the dentinal walls.

The bacterial culture results showed that applying 5.25% NaOCl solution in *E. faecalis* infected root canals, resulted in 99.87% bacterial elimination whereas a 980-nm diode laser with the given parameters resulted in 96.56% bacterial elimination. According to Moritz *et al*. [23], complete eradication of bacteria was achieved only through high-power irradiation of diode laser that raises the temperature on the root surface. Mehrvarzfar *et al*. [31] suggested the combination therapy including chemical irrigation and laser irradiation as an effective treatment option for elimination of *E. faecalis* from the root canal system.

Ashofteh *et al.* [24] observed a 97.56% reduction in the amount of bacteria using a 830-nm diode laser with output power of 1.5 W and claimed that diode laser can be considered as an alternative technique for root canal disinfection. In another study, Rahimi *et al.* [26] reported that laser is less effective in root canal disinfection compared to combined use of laser and NaOCl; hence, using laser in combination with root canal irrigants was recommended. In a similar study, de Souza *et al.* [32] concluded that laser irradiation following chemomechanical irrigation was more effective than NaOCl irrigation alone in root canal disinfection and elimination of *E. faecalis*.

The main difference between the present study and the other similar studies was the method of laser application; in those studies laser irradiation was combined with chemical methods to evaluate its efficacy [22, 32, 33]. This technique is called laser-assisted endodontics. In the present study however, the pure ability of a 980-nm diode laser in *E. faecalis* removal was assessed.

Gutknecht *et al. *[30] demonstrated that the wavelength of 980-nm in diode lasers had the strongest water absorption compared to the other wavelengths of diode lasers and even Nd:YAG. Considering this property of the 980-nm diode laser, most of the laser energy is absorbed in superficial dentinal tubules which is enriched with water. Thus, the superficial dentinal layers receive the most antibacterial effects while the deep dentinal layers receive the less. As a result, the bacteria which penetrate deeply into the dentinal tubules can be secured from laser irradiation and eventually, the disinfection ability of the 980-nm diode laser is reduced. This can also explain the lower disinfection rate in the present study compared to the other similar studies [30].

In addition to the antimicrobial effects, laser application in endodontics could have some other beneficial effects. In the study by Parirokh *et al*. [34] it was showed that application of diode laser after smear layer removal could successfully occlude the dentinal tubules particularly in apical third area which will decrease the risk of reinfection. 

However, application of lasers in root canal disinfection can be accompanied by some limitations and risks such as laser beam not reaching all surfaces, increase in temperature which can lead to surrounding tissue damages and passage of laser beam through the apical foramen and damage to the adjacent anatomic regions. So application of laser devices should be reconsidered [35].

As mentioned before, in this study the pure antibacterial ability of the 980-nm diode laser was assessed. Thus, it is recommended that laser-assisted endodontics (chemical irrigation before laser irradiation) should be used in further studies to evaluate the antibacterial effect of the 980-nm diode laser. It is also recommended that higher output powers and longer irradiation durations would be selected to enhance the efficacy of the 980-nm diode laser.

## Conclusion

The results of the present study showed that 5.25% NaOCl had significantly stronger antibacterial effect compared to a 980-nm diode laser; however, the effectiveness of the 980-nm diode laser in bacterial reduction was acceptable.

## References

[1] Vahdaty A, Ford T, Wilson R (1993). Efficacy of chlorhexidine in disinfecting dentinal tubules in vitro. Dent Traumatol.

[2] Byström A, Sundqvist G (1981). Bacteriologic evaluation of the efficacy of mechanical root canal instrumentation in endodontic therapy. Eur J Oral Sci.

[3] Dalton BC, Ørstavik D, Phillips C, Pettiette M, Trope M (1998). Bacterial reduction with nickel-titanium rotary instrumentation. J Endod.

[4] Nair PR, Sjögren U, Krey G, Kahnberg K-E, Sundqvist G (1990). Intraradicular bacteria and fungi in root-filled, asymptomatic human teeth with therapy-resistant periapical lesions: a long-term light and electron microscopic follow-up study. J Endod.

[5] Berutti E, Marini R, Angeretti A (1997). Penetration ability of different irrigants into dentinal tubules. J Endod.

[6] Kouchi Y, Ninomiya J, Yasuda H, Fukui K, Moriyama T, Okamoto H (1980). Location of Streptococcus mutans in the dentinal tubules of open infected root canals. J Dent Res.

[7] Heling I, Chandler N (1998). Antimicrobial effect of irrigant combinations within dentinal tubules. Int Endod J.

[8] Jeansonne MJ, White RR (1994). A comparison of 2.0% chlorhexidine gluconate and 5.25% sodium hypochlorite as antimicrobial endodontic irrigants. J Endod.

[9] Ørstavik D, Haapasalo M (1990). Disinfection by endodontic irrigants and dressings of experimentally infected dentinal tubules. Dent Traumatol.

[10] Shiozawa A (2000). Characterization of reactive oxygen species generated from the mixture of NaClO and H 2 O 2 used as root canal irrigants. J Endod.

[11] Timpawat S, Vongsavan N, Messer HH (2001). Effect of removal of the smear layer on apical microleakage. J Endod.

[12] Giardino L, Estrela C, Generali L, Mohammadi Z, Asgary S (2015). The in vitro Effect of Irrigants with Low Surface Tension on Enterococcus faecalis. Iran Endod J.

[13] Mohammadi Z, Asgary S (2015). A Comparative Study of Antifungal Activity of Endodontic Irrigants. Iran Endod J.

[14] Estrela C, Estrela C, Decurcio D, Hollanda A, Silva J (2007). Antimicrobial efficacy of ozonated water, gaseous ozone, sodium hypochlorite and chlorhexidine in infected human root canals. Int Endod J.

[15] Haapasalo M, Ørstavik D (1987). In vitro infection and of dentinal tubules. J Dent Res.

[16] Sundqvist G, Figdor D, Persson S, Sjögren U (1998). Microbiologic analysis of teeth with failed endodontic treatment and the outcome of conservative re-treatment. Oral Surg Oral Med Oral Pathol Oral Radiol Endod.

[17] Evans M, Davies J, Sundqvist G, Figdor D (2002). Mechanisms involved in the resistance of Enterococcus faecalis to calcium hydroxide. Int Endod J.

[18] Spratt D, Pratten J, Wilson M, Gulabivala K (2001). An in vitro evaluation of the antimicrobial efficacy of irrigants on biofilms of root canal isolates. Int Endod J.

[19] Brittan J, Sprague S, Huntley S, Bell C, Jenkinson H, Love R (2015). Collagen‐like peptide sequences inhibit bacterial invasion of root dentine. Int Endod J.

[20] Ran S, Gu S, Wang J, Zhu C, Liang J (2015). Dentin tubule invasion by Enterococcus faecalis under stress conditions ex vivo. Eur J Oral Sci.

[21] Weckwerth PH, Zapata RO, Vivan RR, Tanomaru Filho M, Maliza AGA, Duarte MAH (2013). In Vitro Alkaline pH Resistance of Enterococcus faecalis. Brazil Dent J.

[22] Gutknecht N, van Gogswaardt D, Conrads G, Apel C, Schubert C, Lampert F (2000). Diode laser radiation and its bactericidal effect in root canal wall dentin. J Clin Laser Med Surg.

[23] Moritz A, Gutknecht N, Goharkhay K, Schoop U, Wernisch J, Sperr W (1997). In vitro irradiation of infected root canals with a diode laser: results of microbiologic, infrared spectrometric, and stain penetration examinations. Quintessence Int.

[24] Ashofteh K, Sohrabi K, Iranparvar K, Chiniforush N (2014). In vitro comparison of the antibacterial effect of three intracanal irrigants and diode laser on root canals infected with Enterococcus faecalis. Iran J Microbiol.

[25] Kanumuru NR, Subbaiah R (2014). Bacterial Efficacy of Ca (oH) 2 Against E. faecalis Compared with three Dental Lasers on Root Canal Dentin-An Invitro Study. J Clin Diagn Res.

[26] Rahimi S, Shahi S, Gholizadeh S, Shakouie S, Rikhtegaran S, Soroush Barhaghi MH, Ghojazadeh M, Froughreyhani M, Abdolrahimi M (2012). Bactericidal effects of Nd: YAG laser irradiation and sodium hypochlorite solution on Enterococcus faecalis biofilm. Photomed Laser Surg.

[27] Yavari HR, Rahimi S, Shahi S, Lotfi M, Barhaghi MH, Fatemi A, Abdolrahimi M (2010). Effect of Er, Cr: YSGG laser irradiation on Enterococcus faecalis in infected root canals. Photomed Laser Surg.

[28] Figdor D, Davies J, Sundqvist G (2003). Starvation survival, growth and recovery of Enterococcus faecalis in human serum. Oral Microbiol Immunol.

[29] Hubble T, Hatton J, Nallapareddy S, Murray B, Gillespie M (2003). Influence of Enterococcus faecalis proteases and the collagen‐binding protein, Ace, on adhesion to dentin. Oral Microbiol Immunol.

[30] Gutknecht N, Franzen R, Schippers M, Lampert F (2004). Bactericidal effect of a 980-nm diode laser in the root canal wall dentin of bovine teeth. J Clin Laser Med Surg.

[31] Mehrvarzfar P, Saghiri MA, Asatourian A, Fekrazad R, Karamifar K, Eslami G, Dadresanfar B (2011). Additive effect of a diode laser on the antibacterial activity of 2.5% NaOCl, 2% CHX and MTAD against Enterococcus faecalis contaminating root canals: an in vitro study. J Oral Sci.

[32] de Souza EB, Cai S, Simionato MRL, Lage-Marques JL (2008). High-power diode laser in the disinfection in depth of the root canal dentin. Oral Surg Oral Med Oral Pathol Oral Radiol Endod.

[33] Kreisler M, Kohnen W, Beck Myc, Al Haj H, Christoffers A, Götz H, Duschner H, Jansen B, d'Hoedt B (2003). Efficacy of NaOCl/H2O2 irrigation and GaAlAs laser in decontamination of root canals in vitro. Lasers Surg Med.

[34] Parirokh M, Eghbal M, Asgary S, Ghoddusi J, Stowe S, Forghani F, Shahravan A (2006). Effect of 808nm diode laser irradiation on root canal walls after smear layer removal: A scanning electron microscope study. Iran Endod J.

[35] Asnaashari M, Safavi N (2013). Disinfection of Contaminated Canals by Different Laser Wavelengths, while Performing Root Canal Therapy. J Lasers Med Sci.

